# Evaluation of Tolerability, Pharmacokinetics and Pharmacodynamics of Vicagrel, a Novel P2Y12 Antagonist, in Healthy Chinese Volunteers

**DOI:** 10.3389/fphar.2018.00643

**Published:** 2018-06-20

**Authors:** Xiaojiao Li, Cai Liu, Xiaoxue Zhu, Haijing Wei, Hong Zhang, Hong Chen, Guiling Chen, Deming Yang, Hongbin Sun, Zhenwei Shen, Yifan Zhang, Wei Li, Jin Yang, Yongqiang Liu, Xiaojuan Lai, Yanchun Gong, Xuefang Liu, Yongguo Li, Dafang Zhong, Junqi Niu, Bin Liu, Yanhua Ding

**Affiliations:** ^1^Phase I Clinical Trial Unit, First Hospital, Jilin University, Jilin, China; ^2^State Key Laboratory of Drug Research, Shanghai Institute of Materia Medica, Chinese Academy of Sciences, Shanghai, China; ^3^State Key Laboratory of Natural Medicines and Center of Drug Discovery, College of Pharmacy, China Pharmaceutical University, Nanjing, China; ^4^First Hospital and Institute of Immunology, First Hospital, Jilin University, Jilin, China; ^5^Center of Drug Metabolism and Pharmacokinetics, China Pharmaceutical University, Nanjing, China; ^6^Jiangsu Vcare PharmaTech Co. Ltd., Nanjing, China; ^7^Hua Medicine Ltd., Shanghai, China; ^8^Department of Hepatology, First Hospital, Jilin University, Jilin, China; ^9^Department of Hand Surgery, First Hospital, Jilin University, Jilin, China

**Keywords:** vicagrel, pharmacokinetics, pharmacodynamics, safety, clopidogrel

## Abstract

**Background:** Vicagrel is a novel anti-platelet drug and hydrolyzed to the same intermediate as clopidogrel via esterase, instead of CYP2C19. Here we report the first clinical trial on the tolerability, pharmacokinetics and pharmacodynamics of different doses of vicagrel, and comparison with clopidogrel in healthy Chinese volunteers.

**Methods:** This study was conducted in two parts. Study I was a dose-escalating (5–15 mg) study. For each dose, 15 participants were randomized into three groups (total *n* = 45); nine participants were given vicagrel, three were given clopidogrel, and three were given a placebo. Study II was conducted to assess interactions between vicagrel and aspirin in 15 healthy participants. The plasma concentrations of the metabolites of vicagrel and clopidogrel were determined using a LC-MS/MS method. Platelet aggregation was assessed using the VerifyNow-P2Y12 assay.

**Results:** Vicagrel (5–15 mg per day) dosing for 10 days or addition of aspirin was well tolerated in healthy volunteers. The exposure of the active metabolite increased proportionally across the dose range and was higher (~10-fold) than clopidogrel. The levels of IPA dosing 75 mg clopidogrel were between the responses of 5 mg and 10 mg vicagrel. After a single loading dose of vicagrel (30 mg) and a once-daily maintenance dose (7.5 mg) for 8 days, the maximum inhibition of platelet aggregation was similar to that seen with the combined use of vicagrel and aspirin (100 mg/day).

**Conclusion:** Oral vicagrel demonstrated a favorable safety profile and excellent anti-platelet activity, which could be a promising P2Y12 antagonist as anti-platelet drug and can be further developed in phase II/III studies, and marketing for the unmet medical needs of cardiovascular diseases. The study was registered at http://www.chictr.org.cn (ChiCTR-IIR-16009260).

## Introduction

Clopidogrel (Plavix®) is a part of the standard-of-care for patients with acute coronary syndrome (ACS), those undergoing percutaneous coronary intervention (PCI), those with a history of recent myocardial infarction, and following the placement of coronary artery stents (Nguyen et al., 2005; Plosker and Lyseng-Williamson, 2007; Miao et al., 2009; Singh et al., 2010). However, clopidogrel has some limitations called “clopidogrel resistance” because some patients exhibit a low response, no response, or resistance to treatment for cardiovascular diseases (CVDs). These limitations have been of increasing concern in recent clinical practice (Qiu et al., 2013, 2014, 2016). In 2010, the U.S. Food and Drug Administration (FDA) issued a black box warning cautioning that CYP2C19-poor metabolizers (PMs) are at a higher risk of treatment failure with clopidogrel (Shan et al., 2012). Prasugrel (Effient®) has more consistent platelet inhibition than clopidogrel, however, it is associated with an increased risk of bleeding and is generally not recommended among elderly patients (≥75 years old) (Lazar and Lincoff, 2009; Rodriguez et al., 2013; Jeon et al., 2015). Therefore, the U.S. FDA also issued a black box warning for Prasugrel, indicating an increased risk of bleeding resulting from its fast metabolite activation (The specification of EFFIEN, 2009; Qiu et al., 2016). Unlike clopidogrel and prasugrel, ticagrelor (trade name Brilinta®) acts by reversibly binding to the P2Y12 receptor, thus blocking ADP-induced platelet aggregation. Ticagrelor does not require metabolic activation, is quickly absorbed, and exhibits a rapid antiplatelet effect (Anderson et al., 2010; Nawarskas and Clark, 2011; The specification of BRILINT, 2011; Dobesh and Oestreich, 2014). However, an increase in non-procedure-related bleeding, dyspnea, and ventricular pauses have been reported during treatment with ticagrelor (Kowalczyk et al., 2009; Nawarskas and Clark, 2011). Thus, new antiplatelet agents are needed to improve the current antiplatelet therapy.

**Figure 1:**
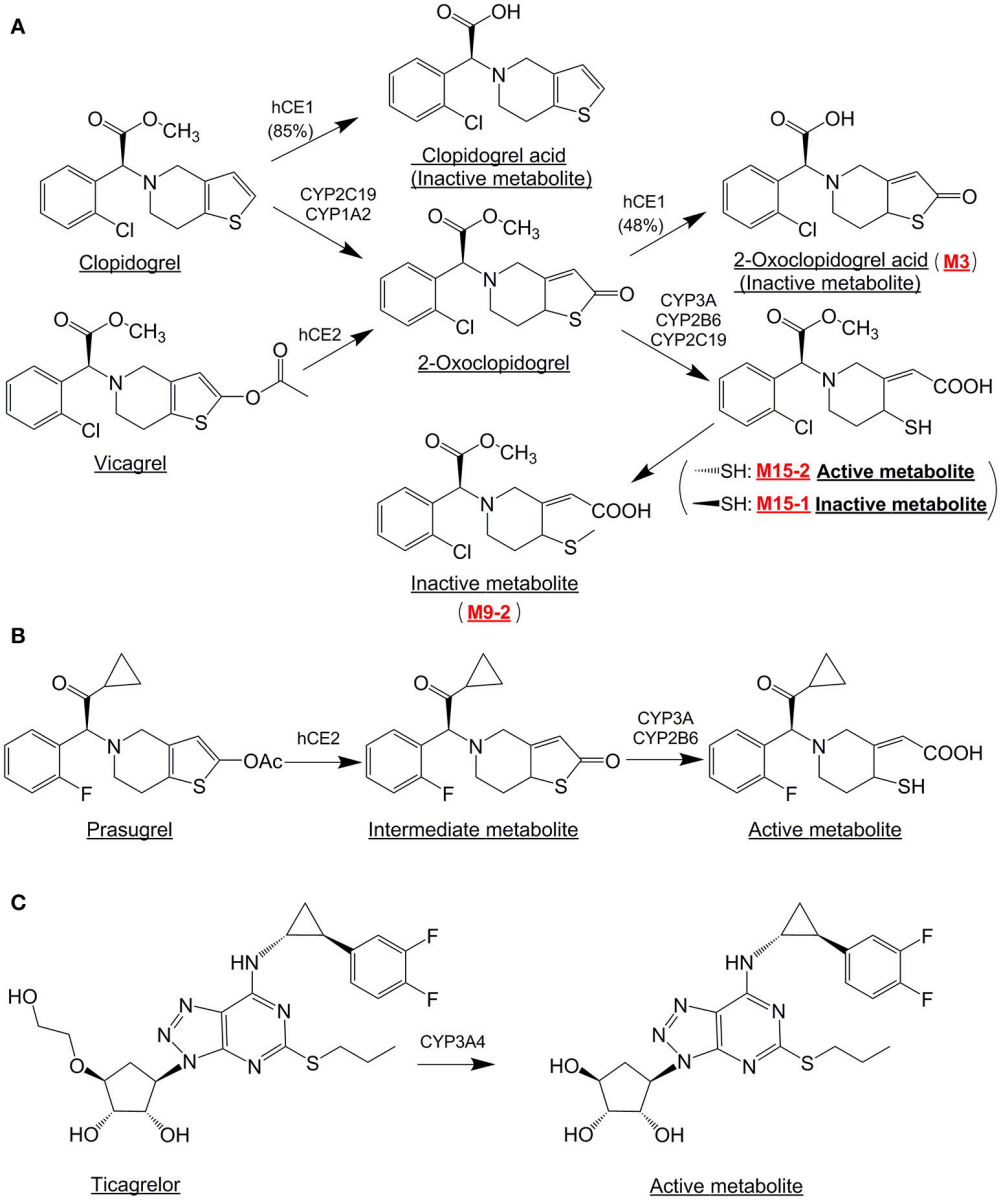
The structures of clopidogrel, vicagrel, prasugrel, ticagrelor, and their active metabolites.

Vicagrel, a novel analog of clopidogrel, has been in clinical development as an agent for antiplatelet therapy. The chemical structures and metabolic pathways of vicagrel, clopidogrel, and other relevant compounds are shown in Figure 1 (Liu et al., 2015). The vicagrel is designed to transform into 2-oxo-clopidogrel, the same intermediate as it from clopidogrel, via hydrolyzed by esterase activity in the body, unlike clopidogrel via CYP2C19 microsomal enzymes (Qiu et al., 2013, 2016). 2-oxo-clopidogrel is further converted into an active metabolite M15-2. It's predicted that dose of vicagrel could be 6–7 times less than that of clopidogrel to reach same level exposure. Based on the above characteristics, vicagrel is expected to have less resistance than clopidogrel, as well as improved safety because the bleeding risk is reduced (Shan et al., 2012; Qiu et al., 2016; Xu et al., 2016).

A number of *in vitro* assay and *in vivo* animal studies in rats and dogs have evaluated the metabolism of vicagrel. Qiu et al. (2016) reported on presystemic bioactivation of vicagrel in the intestinal, hepatic, and prehepatic venous systems of rats, dogs, and humans. In another study in rats, intravenous administration of vicagrel led to rapid conversion to a thiolactone intermediate (2-oxo-clopidogrel) and later to the active metabolite. The transformation efficiency of vicagrel to 2-oxo-clopidogrel was 94% as compared to only 13% for clopidogrel. Oral administration of vicagrel in rats and beagle dogs produced ~6-fold higher exposure of 2-oxo-clopidogrel and 4- to 6-fold higher exposure of the active metabolite than clopidogrel at equimolar doses (Qiu et al., 2013).

The aim of this study was to evaluate the tolerability, the pharmacokinetics (PK) and pharmacodynamics (PD) of vicagrel, meanwhile, to compare with the clopidogrel as control in healthy Chinese participants. These results of Phase I multiple ascending dose (MAD) study will serve as the purpose of dose selection and design of the further clinical study of Phase II in patients. Although this study was not specially designed to evaluate the effect of gene polymorphisms of CYP2C19 on the antiplatelet response of vicagrel, however the individuals were characterized in the presence of its inference.

## Materials and methods

### Participants

The participants enrolled in this study were recruited from Changchun City, China. The inclusion criteria were: healthy individuals of either sex, age ≥ 18 and ≤ 65 years, body weight of at least 50 kg for men and at least 45 kg for women, body mass index ranged from 18.0 to 28.0, voluntary consent to participate in the study, and ability to complete the study in compliance with the protocol. The subjects were instructed to use effective contraception measures during the study period and shouldn't conceive for 6 months after completion of the study. The exclusion criteria were: a clinically significant abnormal laboratory test result or any other significant clinical finding, especially related to any hemorrhagic disease, such as hemorrhoids, acute gastritis, gastric, and duodenal ulcers, thrombocytopenic purpura or hemophilia, etc.; abnormal electrocardiogram, vital signs, or positive findings for hepatitis B virus, hepatitis C virus, or human immunodeficiency virus; donation or loss of >450 mL of blood within the last 3 months; use of any drug known to alter hepatic enzyme activity within 28 days prior to the initial dose of the study medication; use of vitamin supplements, herbal products, prescriptions, or over-the-counter medications within the last 14 days prior to the initial dose of the study medication or during the study; consumption of alcohol within 24 h prior to the initial dose of the study medication; participation in a clinical study in the previous 3 months; and pregnant or lactating females.

### Study design

The study design and clinical protocol were reviewed and approved by the Ethics Committee at the Jilin University First Affiliated Hospital-Clinical Research Institute, Changchun City, China. The clinical trial (registration No.: ChiCTR-IIR-16009260, http://www.chictr.org.cn/) was conducted in accordance with the World Medical Congress Declaration of Helsinki and Good Clinical Practice guidelines. Written informed consent was obtained from all participants. The clinical study was conducted at the Jilin University First Affiliated Hospital-Phase I Clinical Research Center. The drug analysis was performed at the Shanghai Institute of Materia Medica, Chinese Academy of Sciences.

Two separate studies were conducted. Study I was a randomized, double-blind to placebo, single-center, dose-escalating, single and repeat dose study to investigate the safety, tolerability, and PK and PD profiles of vicagrel in healthy Chinese participants. In this study, placebo and clopidogrel were used as negative and positive controls, respectively. Subjects receiving clopidogrel were not blinded in the study. All enrolled participants (*n* = 45) were sequentially assigned to one of three dose-escalating groups: 5, 10, and 15 mg vicagrel, administered once a day. There were 15 participants in each group, out of which 9 participants were administered vicagrel, 3 participants were given clopidogrel (75 mg once daily), and 3 participants were given placebo for 10 days, according to the randomization plan. Study II was conducted to assess the drug-drug interaction between vicagrel and aspirin in healthy Chinese study participants. There were 15 participants in this group. From Day 1 to Day 8, all participants were administered a single oral dose of vicagrel daily. For the next 7 days (Days 9–15), no medication was administered, serving as a 7-day wash-out period. From Day 16 to Day 23, all participants received vicagrel in combination with aspirin. On Day 1 and Day 16, i.e., the first day of each phase, a loading dose of vicagrel (30 mg) was given, and a maintenance dose of vicagrel (7.5 mg) was given for the remainder of the study.

For Study I, 45 random numbers were generated through SAS 9.1 by an independent statistician, and 15 participants were allocated per dose group. In each dose group, participants were randomly assigned to the vicagrel, clopidogrel, or placebo group, according to the proportion of 3:1:1. Forty-five emergency letters containing information about the groups were prepared by the statistician according to the random numbers. Emergency letters were sent to the research center together with the study drug. Study II was an open-label design.

This is a Phase I study of Multiple Ascending Dose (MAD) in health subjects. Its primary goals are to evaluate the tolerability, pharmacokinetics (PK) and pharmacodynamics (PD) of vicagrel, a novel P2Y12 antagonist. Considering the typical design for Phase I MAD contains 8 subjects as one arm, our study design included 15 subjects in each group (9 for vicagrel, 3 for clopidogrel and 3 for placebo) to meet the requirement of Phase I MAD.

### PK analysis

In Study I, serial blood samples (4 mL each) for PK analysis were collected at the following time points: 0 h (pre-dose); 10, 20, 30, and 45 min; and 1, 1.5, 2, 2.5, 3, 4, 6, 8, 12, and 24 h after the first dose; 0 h (pre-dose) on days 7–9; and 0 h (pre-dose) and 10, 20, 30, and 45 min and 1, 1.5, 2, 2.5, 3, 4, 6, 8, 12, 24, 36, 48, and 72 h after dosing on day 10. In Study II, the blood samples were collected at 0 h (pre-dose) and 10, 20, 30, and 45 min and 1, 1.5, 2, 2.5, 3, 4, 6, 8, 12, and 24 h after dosing on day 1; at 0 h (pre-dose) on days 5–7; at 0 h (pre-dose) and 10, 20, 30, and 45 min and 1, 1.5, 2, 2.5, 3, 4, 6, 8, 12, 24, 36, 48, and 72 h after dosing on day 8; and on Day 16, Days 20–23 pre-dose, and at 10, 20, 30, 45 min and 1, 1.5, 2, 2.5, 3, 4, 6, 8, 12, 24, 36, 48, and 72 h after the dose on day 23.

All blood samples for PK analysis were drawn via an indwelling intravenous angiocatheter; the first one milliliter of blood obtained from the catheter was discarded. Derivatization reagent and esterase inhibitor were added to K_2_-EDTA-containing tubes before collection of the blood sample. For the blood samples treated with derivatization agent, 15 μL of 0.5 M 2-bromo-3′-methoxyacetophenone was added before 1.5 mL of blood sample was collected into a K_2_-EDTA-containing tube. For the blood samples treated with esterase inhibitor, 60 μL of 100 mg/mL esterase inhibitor was added before 1.5 mL of blood sample was collected into a K_2_-EDTA-containing tube. These blood samples were centrifuged at 3,500 rpm and 4°C for 5 min after keeping them at room temperature for 10 min. The plasma samples were separated into two polypropylene tubes, and then they were stored at −80°C. For analysis, samples were transported in dry ice.

The plasma concentrations of the metabolites M3, M9-2, M15-1, and M15-2 (active metabolite) of vicagrel and clopidogrel were determined separately using a validated LC-MS/MS method (Liu et al., 2017) after sample preparation.

### PD analysis

Blood samples for platelet aggregation inhibition analysis were collected in 2 mL vacutainer tubes containing 3.2% sodium citrate (VACUETTE, Greiner Bio-One, Australia) via an indwelling intravenous catheter (distinct from the one used for PK sampling). In Study I, the samples were drawn pre-dose on day 1; 4 h after dosing on days 4 and 8; pre-dose on day 10; and 4, 24, 48, and 72 h after dosing on day 10. In Study II, the samples were drawn pre-dose on day 1; 0.5, 1, 2, 4, 8, and 24 h after dosing on day 1; 4 h after dosing on days 3 and 5; pre-dose on day 8; 4, 24, 48, 72, 96, 120, 144, and 168 h after dosing on day 8; pre-dose and 24 h after dosing on day 16; 4 h after dosing on days 18 and 20; pre-dose on day 23; and 4, 24, 48, 72, 96, 120, 144, and 168 h after dosing on day 23.

The VerifyNow® System (Accumetrics, Inc., San Diego, CA, USA) was used to detect inhibition of platelet aggregation (IPA). This turbidimetric-based optical detection system measures platelet aggregation as an increase in light transmittance through whole blood. IPA (%) was calculated from the mathematical formula: IPA (%) = (BASE–PRU)/BASE × 100, where PRU (P2Y12 Reaction Units) reports the amount of P2Y12 receptor mediated aggregation specific to the platelet and are calculated as a function of the rate and extent of platelet aggregation in the ADP channel and BASE is an independent measurement base on the rate and extent of platelet aggregation in the base channel. The BASE result serves as an estimate of the patient's baseline platelet function independent of P2Y12 receptor inhibition. IPA (%) is the percent change from base line aggregation, and is calculated from the PRU result and the BASE result. Two quality controls, electric, and liquid, were used to assure the accuracy of samples.

### Safety assay

Safety and tolerability were evaluated according to the National Cancer Institute Common Terminology Criteria for the Classification of Adverse Events (NCI-CTCAE, 4.03). According to this classification, if more than 50% of the participants experience grade II drug-related adverse events (AEs), more than 25% of the participants had grade III–IV drug-related AEs, or there was one drug-related serious AE, then the treatment group could be considered intolerant. In Study I, safety and tolerability were assessed on days 4, 8, and 13 in each group. In Study II, safety and tolerability were assessed on days 2, 4, 8, 11, 17, 19, 23, and 30.

The tolerability was assessed by monitoring for AEs, vital signs (body temperature, sitting blood pressure, and heart rate, using a HEM-7200 electronic manometer from Omron, Dalian, China), electrocardiograms, physical examination, and clinical laboratory tests (biochemistry tests, hematology tests, urinalysis, coagulation convention examinations, routine stool tests, and occult blood tests). All self-reported AEs were recorded spontaneously by the participants, and the severity and relationship to the study drug were noted for each AE. The electrocardiograms and clinical laboratory tests were conducted at screening and at the completion of the single and multi-dose studies.

### Genotype assay

To genotype CYP2C19, 2 mL of blood were collected from each subject. DNA was extracted from leukocytes using a commercially available kit (Third Wave Technologies, Madison, WI, USA). The genotyping of the mutated genes CYP2C19^*^2 and ^*^3 was conducted using PCR (the restriction fragment length polymorphism method).

### Statistical analysis

The PK parameters of metabolites of vicagrel and clopidogrel were calculated using the non-compartmental method by WinNonLin^®;^, version 6.4 (Pharsight, Mountain View, CA, USA). All statistical tests were performed using SAS 9.2 or later. According to regulatory guidelines, the results were presented as mean values (±standard deviation) or as mean ratios with 90% confidence intervals (CI). The peak time (T_max_) was presented as the median (range). All statistical tests were interpreted at the 5% significance level (two-sided).

## Results

### Baseline characteristics

Of the 60 healthy participants (21 females and 39 males) enrolled in both studies, 45 subjects were included in Study I and the remaining 15 were included in Study II. The demographic and baseline characteristics of all participants are shown in Table 1. The mean age of the 60 participants was 29 ± 6.3 years old, and the mean body weight was 63.6 ± 6.3 kg. Demographic data were similar (*P* > 0.05) across the treatment groups. All participants completed the study. The CYP2C19 genotyping revealed that there were 26 (43.3%) extensive metabolizers (EMs, ^*^1/^*^1), 28 (46.7%) intermediate metabolizers (IMs, ^*^1/^*^2 and ^*^1/^*^3), and 6 (10%) poor metabolizers (PMs, ^*^2/^*^2, ^*^2/^*^3, and ^*^3/^*^3) (Table 1).

**Table 1:** The demographic characteristics (mean ± standard deviation) and CYP2C19 genotyping of participants in this study.

	**Age (y)**	**Body weight (kg)**	**Height (cm)**	**BMI**	**Gender Male [n(%)]**	**Gender Female [n(%)]**	**CYP2C19 EMs [n(%)]**	**CYP2C19 IMs [n(%)]**	**CYP2C19 PMs [n(%)]**
Vicagrel (5 mg; *n* = 9)	30.1 (6.9)	64.3 (10.6)	167.1 (11.3)	23.1 (3.3)	7 (77.8)	2 (22.2)	6 (66.7)	2 (22.2)	1 (11.1)
Vicagrel (10 mg; *n* = 9)	31.9 (5.0)	63.7 (13.0)	161.4 (11.2)	24.3 (2.5)	5 (55.6)	4 (44.4)	3 (33.3)	5 (55.6)	1 (11.1)
Vicagrel (15 mg; *n* = 9)	28.1 (7.0)	65.5 (10.9)	166.6 (9.8)	23.5 (2.6)	5 (55.6)	4 (44.4)	2 (22.2)	6 (66.7)	1 (11.1)
Clopidogrel (*n* = 9)	30.3 (7.5)	63.8 (8.5)	165.4 (7.2)	23.4 (3.2)	6 (66.7)	3 (33.3)	5 (55.6)	4 (44.4)	0 (0)
Placebo (*n* = 9)	27.6 (7.5)	61.3 (7.7)	168.2 (8.2)	21.6 (1.9)	6 (66.7)	3 (33.3)	4 (44.4)	5 (55.6)	0 (0)
Vicagrel (loading dose of 30 mg and maintenance dose of 7.5 mg + aspirin at 100 mg) (*n* = 15)	27.3 (4.4)	63.1 (8.7)	164.5 (9.5)	23.3 (2.8)	10 (66.7)	5 (33.3)	6 (40.0)	6 (40.0)	3 (20.0)
*P* (difference between 6 groups)	0.5339	0.9312	0.6488	0.3450	0.8964		0.5967		
Total (*n* = 60)	29.0 (6.3)	63.6 (6.3)	165.4 (9.5)	23.2 (2.7)	39 (65.0)	21 (35.0)	26 (43.3)	28 (46.7)	6 (10.0)

*BMI, body mass index; EMs, extensive metabolizers; IMs, intermediate metabolizers; PMs, poor metabolizers; n, sample size*.

*Statistically significance, P < 0.05*.

### PK profile of the active and inactive metabolites of vicagrel and clopidogrel

**Figure 2:**
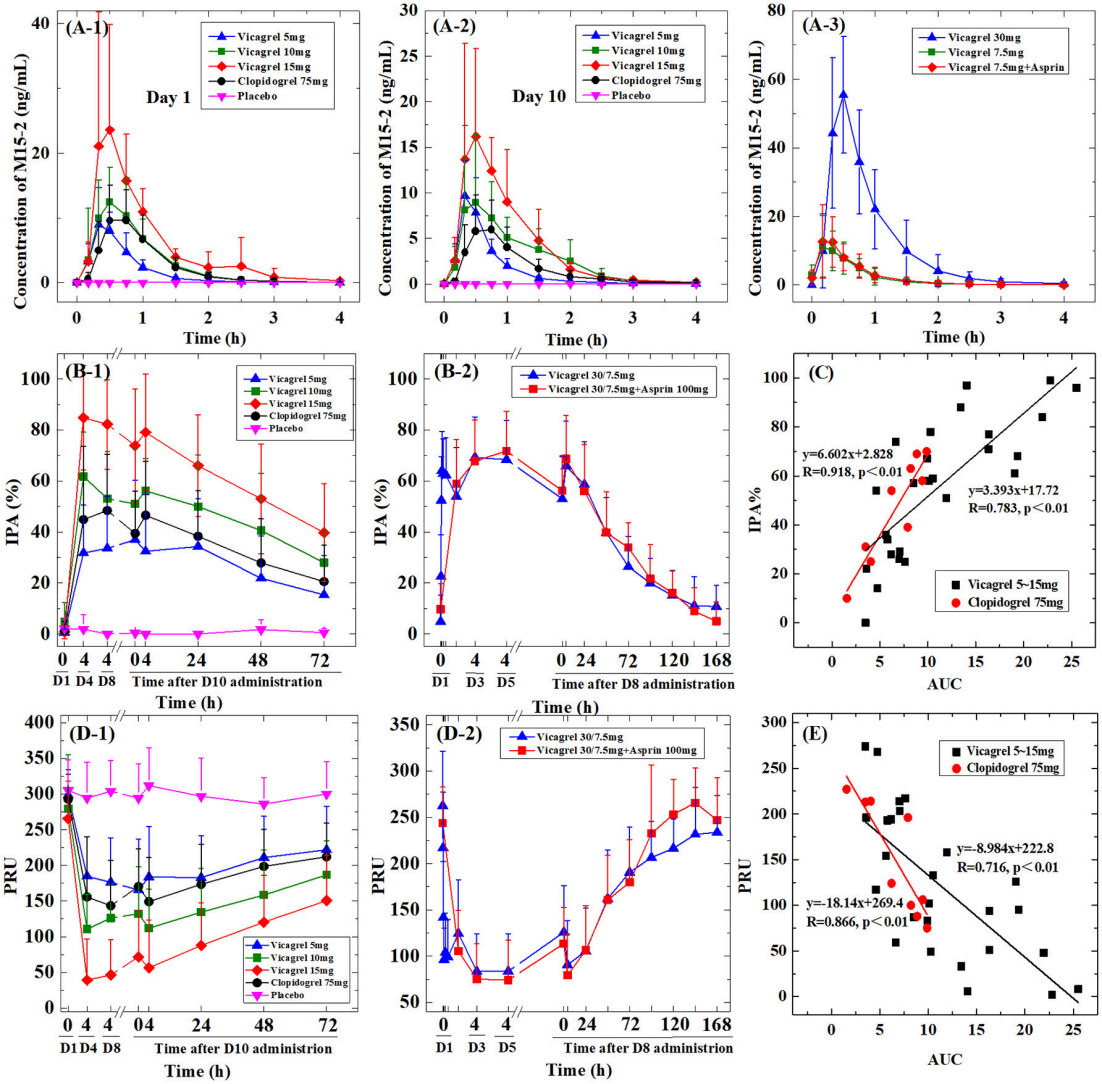
The vicagrel and clopidogrel PK, PD profiles and the PK-PD relationship. **(A-1)** The M15-2 PK profiles of vicagrel (5 mg, 10 mg, or 15 mg), clopidogrel (75 mg), and placebo on Day 1, **(A-2)** Day 10 and **(A-3)** The M15-2 PK profiles of vicagrel (30 mg, 7.5 mg, or 7.5 mg combined with aspirin at 100 mg/day). **(B-1)** The mean IPA% of vicagrel, clopidogrel, and placebo treatment groups and **(B-2)** vicagrel at a single loading dose of 30 mg followed by a once-daily maintenance dose of 7.5 mg, with or without aspirin at 100 mg/day. **(C)** the relationship between the IPA% on Day 10 at 4 h post-administration, and the AUC in the vicagrel and clopidogrel treatment groups. **(D-1)** The mean PRU of vicagrel, clopidogrel, and placebo treatment groups and **(D-2)** vicagrel at a single loading dose of 30 mg followed by a once-daily maintenance dose of 7.5 mg, with or without aspirin. And **(E)** the relationship between the PRU on Day 10 at 4 h post-administration, and the AUC in the vicagrel and clopidogrel treatment groups.

All enrolled participants were evaluated for PK parameters. The area under the curve (AUCs) of the metabolites (M3, M9-2, M15-1, and M15-2) of vicagrel and clopidogrel are summarized in Table 2. The mean plasma concentration-time profiles of the active metabolites of vicagrel and clopidogrel (M15-2) in Study I are shown in Figures 2A-1,A-2). After 10 days of daily administration, all the metabolites of vicagrel and clopidogrel reached their steady states, and there was no significant accumulation with multiple doses (accumulation factor of ~1). The plasma concentration of the active metabolite of vicagrel increased rapidly and reached its peak at 0.33–0.50 h, and the clopidogrel active metabolite reached its peak at 0.75 h, after drug administration on both day 1 and day 10. The C_max_ and AUC of the active metabolite increased proportionally to the dose of vicagrel from 5 to 15 mg. At steady state, the PK parameters of the active metabolite of clopidogrel (75 mg) were similar to those of the active metabolite formed with the administration of vicagrel (5 mg).

**Table 2:** The area under the curve for the active metabolite (M15-2) and inactive metabolites (M15-1, M3, and M9-2) of each treatment group (ng · h/mL).

	**Study I**								**Study II**		
	**Day 1**				**Day 10**				**Day 1**	**Day 8**	**Day 23**
	**Vicagrel (5 mg)**	**Vicagrel (10 mg)**	**Vicagrel (15 mg)**	**Clopidogrel (75 mg)**	**Vicagrel (5 mg)**	**Vicagrel (10 mg)**	**Vicagrel (15 mg)**	**Clopidogrel (75 mg)**	**Vicagrel (30 mg)**	**Vicagrel (7.5 mg)**	**Vicagrel (7.5 mg** + **aspirin at 100 mg)**
M15-2	6.23 (2.34)	12.2 (4.35)	22.5 (10.1)	9.83 (4.42)	5.84 (2.00)	11.1 (4.46)	16.7 (6.03)	6.50 (2.98)	47.9 (17.8)	10.3 (4.45)	11.7 (4.77)
M15-1	9.00 (3.49)	18.3 (7.07)	30.2 (4.48)	9.68 (3.67)	9.66 (2.57)	19.4 (7.11)	28.1 (6.49)	9.26 (4.36)	64.0 (17.2)	16.0 (4.84)	19.1 (5.78)
M3	81.5 (16.4)	216 (107)	370 (85.4)	270 (81.4)	108 (33.7)	221 (80.2)	427 (131)	292 (82.6)	672 (183)	151 (29.9)	152 (28.0)
M9-2	808 (410)	1,420 (378)	2,110 (539)	795 (279)	958 (286)	1,740 (663)	2,720 (954)	946 (418)	3,680 (697)	1,590 (493)	1,720 (507)

**Figure 3:**
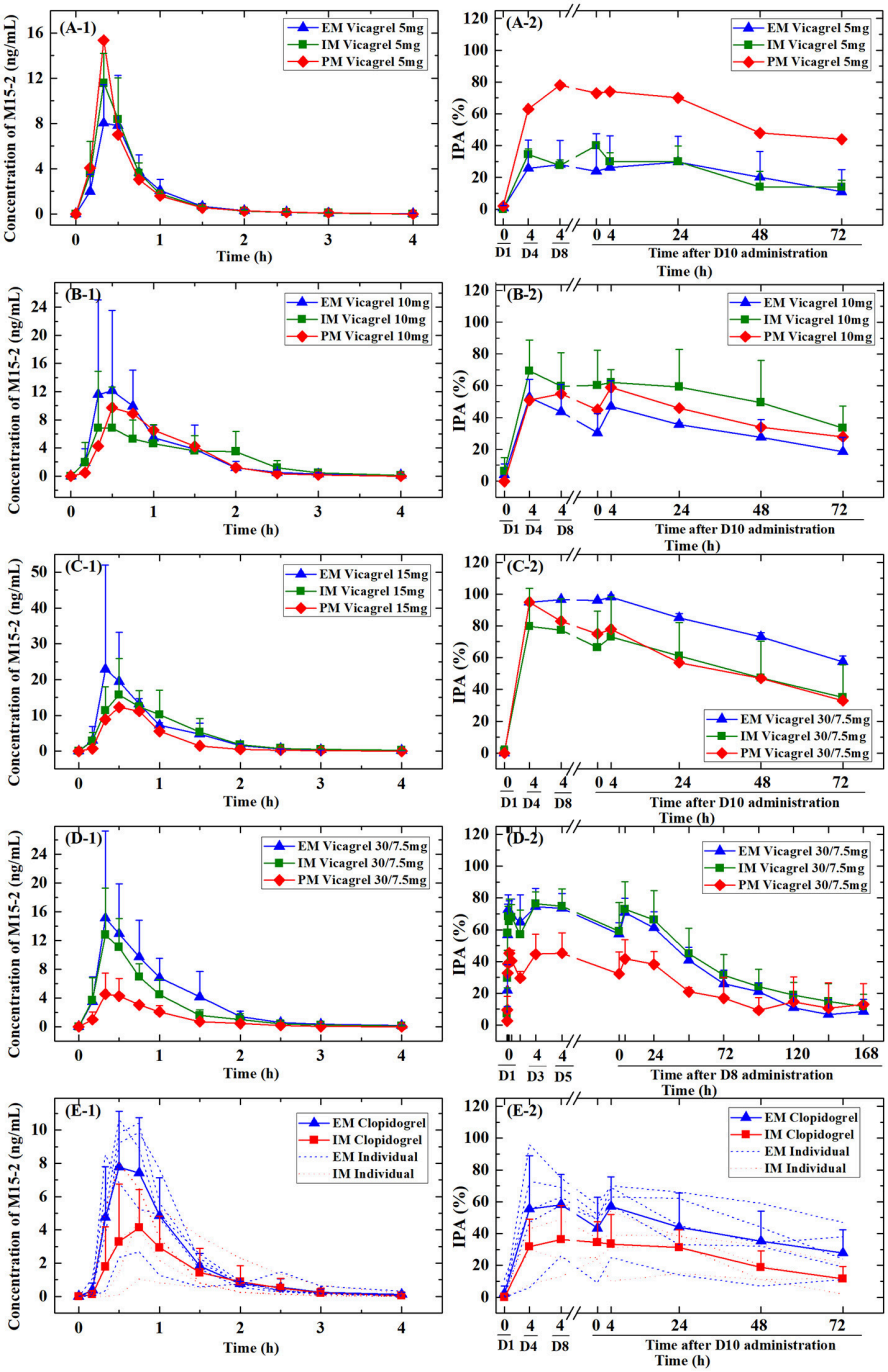
Plasma concentrations of active metabolites and the corresponding inhibition of platelet aggregation (IPA%) of vicagrel and clopidogrel stratified by CYP2C19 polymorphism for each treatment group. EM, extensive metabolizers; IM, intermediate metabolizers; PM, poor metabolizers. **(A-1,B-1,C-1,D-1,E-1)** The M15-2 PK profiles of vicagrel (5, 10, 15, 30/7.5 mg) and clopidogrel (75 mg), **(A-2,B-2,C-2,D-2,E-2)** The IPA% of vicagrel (5, 10, 15, 30/7.5 mg) and clopidogrel (75 mg).

The plasma concentration of M15-2 peaked ~0.50 h after administration of the loading dose on day 1, and after the maintenance dose on day 8, and when combined with aspirin (day 23) in the group receiving a loading dose of 30 mg and a maintenance dose of 7.5 mg. The PK parameters of all four metabolites were similar between the vicagrel maintenance (7.5 mg) group and the combination (with aspirin) group (90% CI: 82.46–137.33%), indicating that there were no drug-drug interactions between vicagrel and aspirin. The mean plasma concentration-time profiles of the vicagrel active metabolite (M15-2) in Study II are shown in Figure 2A-3.

All other metabolites (M15-1, M3, and M9-2) peaked quickly (0.50–1.50 h), and, except for M9-2 (t_1/2_, ~12 h), all others were also eliminated quickly. In addition, M9-2 had the highest system exposure; the AUC was about 128–135-fold higher than that of the active metabolite M15-2. The PK characteristics were similar for M15-1 and M15-2. All metabolites displayed linear PK properties. Lastly, there were no significant differences in PK parameters between men and women, indicating that gender does not affect the PK of vicagrel and clopidogrel.

### PD evaluation

The PD parameters were directly assessed using an Accumetrics VN-P2Y_12_ assay. In Study I, after treatment for 10 days, vicagrel led to inhibition of platelet aggregation (IPA) in a dose-dependent manner; 32.4% inhibition with 5 mg, 60.7% with 10 mg, and 79.1% with 15 mg at 4 h after dosing on day 10. There was 46.6% inhibition at 4 h after the dose on day 10 in the clopidogrel treatment group; and this an inhibition level lay between those seen with the 5 and 10 mg doses of vicagrel. Furthermore, the IPA by clopidogrel in CYP2C19 IMs was significantly lower than that in the EMs. However, the IPA by vicagrel was not statistically related to CYP2C19 polymorphisms.

In Study II, participants were administered a single loading dose (30 mg) of vicagrel, followed by once-daily maintenance doses (7.5 mg) for 8 days. In this group, there was 65.8% inhibition at 4 h after dosing on Day 8. This result was similar to that seen with the combined use of vicagrel and aspirin (68.7%). IPA after the maintenance dose was maintained at the same level as that seen after the loading dose.

The P2Y12 Reaction Units (PRU) as determined in the VerifyNow assay was rapidly reduced by vicagrel and clopidogrel, reaching maximum inhibition 4 h after two drugs administration. The PRU was 149.2 at 4 h after the dose on day 10 in the clopidogrel treatment group; and this PRU level lay between those seen with the 5 mg and 10 mg doses of vicagrel. The PRU results showed similar trends to those calculated for IPA.

A summary of the IPA data obtained from Study I and Study II is presented in Table 3. The mean PD results of all sampling times from Day 1–10 in Study I, and Day 1–23 in Study II are presented in Figures 2B-1,B-2,D-1,D-2. The mean IPA and PRU values of clopidogrel were between the IPA and PRU seen with the 5 mg and 10 mg doses of vicagrel across all time points.

**Table 3:** Inhibition of platelet aggregation (%) over time in Study I and Study II.

**Day**	**Time (h)**	**IPA(%)**						
		**Vicagrel (5 mg; *N* = 9)**	**Vicagrel (10 mg; *N* = 9)**	**Vicagrel (15 mg; *N* = 9)**	**Clopidogrel (75 mg; *N* = 9)**	**Placebo (*N* = 9)**	**Vicagrel (30 mg/7.5 mg; *N* = 15)**	**Vicagrel (30 mg/7.5 mg + aspirin at 100 mg; *N* = 15)**
1	0	0.67 (1.4)	4.89 (7.3)	0.89 (2.7)	1.22 (3.7)	2.11 (4.2)	4.9 (10.4)	9.7 (10.1)
1	0.5	–	–	–	–	–	22.5 (16.4)	–
1	1	–	–	–	–	–	50.4 (20.1)	–
1	2	–	–	–	–	–	64.1 (15.2)	–
1	4	–	–	–	–	–	62.9 (13.6)	–
1	8	–	–	–	–	–	62.4 (14.6)	–
1	24	–	–	–	–	–	53.9 (19.2)	58.9 (17.3)
3	4	–	–	–	–	–	69.3 (15.8)	71.2 (15.0)
4	4	31.8 (18.8)	59.7 (18.0)	84.8 (20.5)	44.9 (28.7)	1.9 (5.7)	–	–
5	4	–	–	–	–	–	68.4 (15.4)	71.7 (15.6)
8	0	–	–	–	–	–	53.0 (16.6)	56.2 (13.7)
8	4	33.6 (20.5)	53.1 (18.5)	82.2 (17.6)	48.4 (22.1)	0.0 (0.0)	65.8 (17.7)	68.7 (17.1)
8	24	–	–	–	–	–	58.6 (16.8)	56.1 (18.3)
8	48	–	–	–	–	–	39.7 (13.9)	39.8 (16.0)
8	72	–	–	–	–	–	26.4 (11.9)	32.1 (11.7)
8	96	–	–	–	–	–	19.9 (9.8)	21.7 (13.3)
8	120	–	–	–	–	–	15.1 (9.8)	16.2 (8.5)
8	144	–	–	–	–	–	11.1 (11.3)	8.9 (9.3)
8	168	–	–	–	–	–	10.8 (8.3)	5.0 (7.6)
10	0	37.0 (23.3)	51.0 (22.0)	73.9 (22.2)	39.4 (16.7)	0.4 (1.1)	–	–
10	4	32.4 (22.2)	60.7 (18.1)	79.1 (22.8)	46.6 (21.2)	0.0 (0.0)	–	–
10	24	34.3 (18.8)	50.0 (20.3)	66.0 (19.9)	38.3 (17.9)	0.0 (0.0)	–	–
10	48	21.9 (16.6)	40.6 (22.4)	53.0 (21.5)	27.9 (17.2)	0.8 (1.4)	–	–
10	72	15.3 (15.6)	28.0 (12.9)	39.8 (19.1)	20.6 (14.2)	0.8 (1.7)	–	–

*Values are given as the mean (standard deviation)*.

### PK-PD relationship

Next, the relationship between the anti-platelet effects and the AUC was investigated. A significant correlation was observed between the AUC and the maximum IPA%/minimal PRU on day 10 at 4 h after dosing (*P* < 0.01). This trend was observed in both the vicagrel and clopidogrel groups (Figures 2C,E).

### Pharmacogenetic analysis

The plasma concentrations of the active metabolites of vicagrel and clopidogrel were evaluated according to stratification by CYP2C19 polymorphisms present in each treatment group (Figure 3). The AUC of the active metabolite of clopidogrel differed significantly between EMs and IMs. The C_max_ among IMs was also significantly lower than that of EMs (there were no PMs in the clopidogrel group). The effect of the CYP2C19 polymorphism on the PK parameters of vicagrel was not significant in the 5 and 10 mg groups; however, PMs had lower concentrations of the active metabolite than did IMs or EMs for both the 15 and 7.5 mg maintenance dose groups.

Among vicagrel-treated participants, the CYP2C19-predicted phenotype group was not statistically associated with an increased or decreased anti-platelet effect. However, among clopidogrel-treated participants, the CYP2C19-predicted phenotype group was consistently associated with a dampened PD effect among EMs and IMs for all platelet aggregation measures (Figure 3).

### Safety and tolerability

Vicagrel treatment for 10 days at 5–15 mg per day was safe and well tolerated in healthy Chinese study participants. The other regimen, a loading dose of 30 mg of vicagrel, followed by 7.5 mg of vicagrel + 100 mg of aspirin daily for 8 days, was also safe and well tolerated. The summary of adverse events is shown in Table 4. Most AEs were mild in severity (CTCAE grade 1), and there was no reporting of serious AEs. Three grade 2 AEs i.e., orchitis (placebo), increased alanine aminotransferase levels and diarrhea (vicagrel 10 mg) were reported. The most frequently reported AEs were mild bleeding, ecchymosis (≤26.7%), hemorrhage (≤22.2%), and bleeding gums (≤13.3%). For all these events, the incidence was higher in the vicagrel plus aspirin group than in the other treatment groups. No abnormal coagulation test results were reported throughout the study, except for one case in the placebo group. No participants withdrew from the study because of AEs, and no abnormal changes in vital signs or electrocardiography were reported.

**Table 4:** Adverse events reported in the study arms.

**Adverse Events**	**Study I**					**Study II**	
	**Vicagrel (5 mg; *N* = 9)**	**Vicagrel (10 mg; *N* = 9)**	**Vicagrel (15 mg; *N* = 9)**	**Clopidogrel (75 mg; *N* = 9)**	**Placebo (*N* = 9)**	**Vicagrel (30 mg/7.5 mg; *N* = 15)**	**Vicagrel (30 mg/7.5 mg + aspirin at 100 mg; *N* = 15)**
Total	6 (66.7%)	5 (55.6%)	4 (44.4%)	1 (11.1%)	7 (77.8%)	3 (20.0%)	11 (73.3%)
*P* (Compared with clopidogrel)	0.0498^*^	0.1312	0.2941	NA	0.0152^*^	–	–
*P* (Compared with placebo)	1.0000	0.6199	0.3348	0.0152^*^	NA	–	–
*P* (Compared with DDI group)	–	–	–	–	–	0.0092^**^	NA
**METABOLISM AND NUTRITION DISORDERS**							
Hypokalemia	0	0	0	0	1 (11.1%)	0	0
Hypoalbuminemia	0	0	0	0	0	1 (6.7%)	0
**INFECTIONS AND INFESTATIONS**							
Urinary tract infection	0	1 (11.1%)	0	0	0	0	0
Orchitis	0	0	0	0	1 (11.1%)	0	0
**INVESTIGATIONS**							
Increased alanine aminotransferase	0	1 (11.1%)	1 (11.1%)	0	2 (22.2%)	1 (6.7%)	0
Increased international normalized ratio increased	0	0	0	0	1 (11.1%)	0	0
Present protein urine	0	1 (11.1%)	0	0	0	0	0
Decreased retinol-binding protein	0	1 (11.1%)	0	0	0	0	0
Increased aspartate aminotransferase increased	0	1 (11.1%)	0	0	1 (11.1%)	1 (6.7%)	0
Increased blood glucose	1 (11.1%)	0	0	0	0	0	0
Increased white blood cell count increased	0	0	0	0	0	1 (6.7%)	0
Increased total bilirubin	0	0	0	0	0	1 (6.7%)	2 (13.3%)
Increased indirect bilirubin	0	0	0	0	0	1 (6.7%)	2 (13.3%)
Increased total bile acid	0	0	0	0	0	0	1 (6.7%)
**NERVOUS SYSTEM DISORDERS**							
Scalp hypoesthesia	1 (11.1%)	0	0	0	0	0	0
**RESPIRATORY, THORACIC, AND MEDIASTINAL DISORDERS**							
Epistaxis	1 (11.1%)	0	2 (22.2%)	0	0	0	3 (20.0%)
**SKIN AND SUBCUTANEOUS TISSUE**							
Rash	1 (11.1%)	0	0	0	1 (11.1%)	0	0
Ecchymosis	0	0	1 (11.1%)	0	0	0	4 (26.7%)
Pruritus	1 (11.1%)	0	0	1 (11.1%)	0	0	0
**GENERAL DISORDERS AND ADMINISTRATION SITE CONDITIONS**							
Chest discomfort	0	0	0	0	1 (11.1%)	0	0
Fever	0	0	0	0	0	1 (6.7%)	0
**GASTROINTESTINAL DISORDERS**							
Bleeding gums	0	0	0	0	1 (11.1%)	0	2 (13.3%)
Diarrhea	1 (11.1%)	1 (11.1%)	0	0	0	0	0
**CARDIAC DISORDERS**							
Ventricular premature beat	1 (11.1%)	0	0	0	0	0	0
**BLOOD AND LYMPHATIC SYSTEM**							
Anemia	0	0	0	0	0	0	1 (6.7%)

*Statistical significance*,

* *P < 0.05*,

** *P < 0.01*.

## Discussion

The results from this study show that vicagrel was rapidly and entirely absorbed and converted into its intermediate and then to its active metabolites. The T_max_ of the active metabolite occurred at 0.33–0.50 h after dosing. Vicagrel was well tolerated in the dose range of 5–15 mg/day and when used in combination with aspirin at 100 mg/day. This rapid absorption and activation was similar to that of prasugrel (Cui et al., 2012) and faster than that of clopidogrel (~0.75 h), which is one of most advantages in clinical to benefit for the emergency care of heart disease.

Vicagrel led to a higher (~10-fold or more) exposure of the active metabolite than clopidogrel. Exposure to the vicagrel active metabolite increased in a dose-dependent manner from 5 to 15 mg. There were no significant changes in the PK parameters of vicagrel on combined use with aspirin at 100 mg/day.

M15-1 and M15-2 are enantiomers, and M15-2 is the common active metabolite of clopidogrel and vicagrel. The majority of the previously published studies have reported the total amount of both the enantiomers. The two enantiomers were analyzed separately in the current study. As a result, the M15-2 concentrations reported in this study were slightly lower than the active metabolite concentrations reported in previous studies (Umemura and Iwaki, 2016).

The PD parameters assessed by the VN-P2Y12 assay show that IPA by vicagrel occurred in a dose-dependent manner, with 32.4–79.1% inhibition at doses of 5–15 mg (at 4 h after dosing). The levels of IPA by clopidogrel at a dose of 75 mg were between the responses observed with the 5 and 10 mg doses of vicagrel. The drug-drug interaction (DDI) results showed stable platelet aggregation inhibition by vicagrel alone and when used in combination with aspirin. Addition of aspirin had no effect on the IPA value as compared to vicagrel alone. In a previous study, the IPA by prasugrel 20 mg was significantly greater than that seen with clopidogrel 75 mg dose (Umemura and Iwaki, 2016). The IPA with vicagrel 15 mg in our study was similar to that seen with prasugrel 20 mg (Umemura and Iwaki, 2016) and ticagrelor 180 mg (Jeon et al., 2015).

According to the DRUG LABEL INFORMATION of clopidogrel^1^, at steady state, the average inhibition level observed with a dose of 75 mg clopidogrel per day was between 40% and 60% (DRUG LABEL INFORMATION of clopidogrel^1^). And according to another study, the mean IPA was 55% for clopidogrel 75 mg once daily group (Vrijens et al., 2014). PRU < 208 at 12 to 24 h after percutaneous coronary intervention or during follow-up was associated with a lower risk for cardiovascular events when treatment with clopidogrel (Price et al., 2011). High on-treatment platelet reactivity was defined as 208 or more PRU and low on-treatment reactivity was defined as 85 or less PRU; these cut off values have been associated with more ischaemic and bleeding events, respectively (Cayla et al., 2016). These data are similar with the results of our study. And the IPA and PRU results for 10 mg vicagrel was on the same or slightly higher level of 75 mg clopidogrel.

The active metabolite of vicagrel and clopidogrel has irreversible effects on P2Y12 on the platelet membrane. However, the life of circulating human platelets is only 8–10 days, and ~20% platelets are renewed daily (Umemura et al., 2017). After the discontinuation of the vicagrel and clopidogrel, the blood platelets will gradually back to normal in 5–8 days (DRUG LABEL INFORMATION of clopidogrel^1^; Figure 2).

The genetic evaluation conducted in this study confirmed previous observations that CYP2C19 phenotype plays an important role in the variability of response to clopidogrel (Miao et al., 2009; Shuldiner et al., 2009). Despite the small sample size of the current study, the data is consistent with the design rationale that the efficacy of vicagrel might not be affected by CYP2C19 polymorphisms. This indicated that vicagrel could be used among all CYP2C19 genotype patients as compared to selective usage of clopidogrel due to “clopidogrel resistance.” Theoretically, vicagrel is hydrolyzed to its intermediate directly and rapidly by the involvement of carboxyl esterases, rather than CYP2C19 (only about 10% contribution to 2-oxoclopidogrel metabolism) (Djebli et al., 2015), during the absorption phase before entry into the liver (Taketani et al., 2007; Hosokawa, 2008; Williams et al., 2008; Imai and Ohura, 2010; Hagihara et al., 2011; Qiu et al., 2014). Because of the highly variable PK and PD results for vicagrel in all treatment groups, the carboxyl esterase-related polymorphisms should be investigated in future studies. And except for the occurrence of SNPs, the variable response to antiplatelet agents may relate to different non-genetic factors when administered to patients, such as demographic characteristics (age, BMI, smoking…), concurrent diseases, and drug interactions (Zhang et al., 2017). These factors will be considered in further studies among patients.

In conclusion, the purpose of the present study is to investigate the PK, PD, and tolerability of vicagrel, a novel P2Y12 antagonist in healthy Chinese subjects. All the doses (5, 10, and 15 mg) of vicagrel were generally well tolerated. Dosing with vicagrel led to greater exposure to the active metabolite and more consistent PD responses across all groups as compared to clopidogrel. As a novel P2Y12 antagonist, vicagrel shows its advantage in following characterization, such as well tolerable, quicker absorption and therapy, ~10-fold less dosing and patient economical, comparing to clopidogrel, the current standard treatment agent. In further studies, the clinical efficiency of vicagrel will be evaluated in patients that receive anti-platelet therapy.

## Author contributions

XJLi and YD wrote the article. YD and HS designed the research. XJLi, XZ, HW, HZ, HC, GC, DY, ZS, JN, BL, and YD performed the research. CL, YZ, WL, and DZ analyzed the data. HS, JY, YQL, XJLa, YG, XFL, and YGL contributed the new reagents.

### Conflict of interest statement

The authors declare that the research was conducted in the absence of any commercial or financial relationships that could be construed as a potential conflict of interest.

## Footnotes

^1^DRUG LABLE INFORMATION of clopidogrel. Available online at: https://dailymed.nlm.nih.gov/dailymed/search.cfm?query=clopidogrel
